# Concentrations of Carotenoids and Tocopherols in Breast Milk from Urban Chinese Mothers and Their Associations with Maternal Characteristics: A Cross-Sectional Study

**DOI:** 10.3390/nu9111229

**Published:** 2017-11-09

**Authors:** Yong Xue, Esther Campos-Giménez, Karine Meisser Redeuil, Antoine Lévèques, Lucas Actis-Goretta, Gerard Vinyes-Pares, Yumei Zhang, Peiyu Wang, Sagar K. Thakkar

**Affiliations:** 1 Department of Nutrition & Food Hygiene, School of Public Health, Peking University Health Science Center, No. 38 Xueyuan Road, Haidian District, Beijing 100191, China; xueyong_pku@bjmu.edu.cn (Y.X.); wpeiyu138@163.com (P.W.); 2 Nestlé Research Center, Nestec, Vers-chez-les-blanc, CH-1000 Lausanne 26, Switzerland; Karine.Meisser@rdls.nestle.com (K.M.R.); Antoine.Leveques@rdls.nestle.com (A.L.); lucas.actisgoretta@rdls.nestle.com (L.A.-G.); sagar.thakkar@rdls.nestle.com (S.K.T.); 3 Nestlé Research Center Beijing, Building E-F, No. 5 Dijin Road, Haidian District, Beijing 100095, China; Gerard.VinyesPares@nestle.com

**Keywords:** breast milk, carotenoids, tocopherols, colostrum, lactation stage, cross-sectional study

## Abstract

Milk composition remains the best estimate of infant requirements. The aims of this study were to quantify carotenoids and tocopherols in human milk from healthy Chinese mothers, and to explore their associations with lactation stage, region, socio-economic and obstetric characteristics, and dietary intake. Human milk was obtained from 509 healthy mothers, and concentrations of carotenoids and tocopherols were analyzed by Ultra High Performance Liquid Chromatography. The mothers’ socio-economic and obstetric characteristics and dietary intake through a single 24-h dietary recall were evaluated. The median concentrations (μg/100 mL) of each component of 0–4 days, 5–11 days, 12–30 days, 31–60 days, 61–120 days, and 121–240 days *postpartum* were respectively as follows: β-carotene 8.0, 2.8, 2.1, 1.7, 1.9, 1.8; β-cryptoxanthin 6.2, 3.4, 2.4, 1.7, 1.8, 2.1; lutein 5.7, 7.0, 2.2, 2.9, 2.8, 3.7; lycopene 6.3, 2.5, 1.8, 1.4, 1.4, 1.5; zeaxanthin 1.0, 1.4, 0.8, 0.8, 1.0, 1.1; α-tocopherol 645, 382, 239, 206, 212, 211; γ-tocopherol 68, 63, 70, 73, 68, 88. The levels of those components varied significantly among different lactation stages and presented regional differences. Associations of carotenoid contents with maternal education, delivery mode, and present body mass index were found in multivariate analyses. These results suggested that lactation stage, region, and socio-economic and obstetric factors were associated with human milk concentrations of carotenoids and tocopherols in healthy Chinese mothers.

## 1. Introduction

According to the World Health Organization, exclusive breastfeeding is recommended for the first six months of life [1], the period within which breast milk is the sole source of nutrition, providing all necessary nutrients to maintain health and permit normal growth. Thereafter, complementary feeding should be introduced while breastfeeding continues up to two years of age or beyond, so that breast milk is still a significant source of nutrients, at least in some parts of the world [2]. The recommended micronutrient intake for infants is currently based on the amounts provided by human milk from well-nourished women [3,4]; although this has been questioned by some authors, given the high variability observed among individuals [5,6]. Vitamin A is present in breast milk in the forms of preformed retinol (as retinyl esters), but also present as provitamin A carotenoids (α-carotene, β-carotene and β-cryptoxanthin). Since infants are born with very low reserves of vitamin A in the liver regardless of the mother’s nutritional status [3], they rely entirely on breast milk to support growth and build up liver storage. Vitamin E is a family of eight naturally occurring compounds sharing a common structure, and breast milk contains primarily α-tocopherol, followed by smaller amounts of γ- and β-tocopherol. Vitamin A and vitamin E are required in many essential metabolic functions for the growing infant [7,8].

Given the importance of all these compounds for the newborn and the specific risk of fat-soluble vitamin deficiency in lactating women and breastfed children [9,10], the need to determine their concentrations in breast milk and how they depend on external or internal factors has been identified. A large number of reports in the composition of milk from Chinese mothers have focused on macronutrients [11,12,13], minerals [11,14], and fatty acids [14,15,16,17,18,19,20], while only a few studies report fat-soluble vitamin or carotenoid values [21,22,23]. Shi et al. [22] provide data on the vitamin content of milk from Chinese mothers from Inner Mongolia, which might be difficult to extrapolate to the whole country, partially due to the low number of data points and partially because it represents only a part of the country. The multinational study of Canfield et al. [21] showed differences in carotenoid patterns between countries, reflecting that in each one, the dietary carotenoid supply had no indication on the geographical origin of all samples, although this might be limited. Another multinational study [23] showed that regardless of the difficulty to detect trends due to high individual variability, some carotenoids presented clear differences between countries, which confirmed previous results, but also presented the same drawbacks related to the limited geographical variability of the samples. Thus, considering the geographical extension and multivariate lifestyles and diet in different parts of China, it would be necessary to study the carotenoid and tocopherol compositions of milk from Chinese women from different regions through lactation.

The changes in milk composition depending on different factors, such as stage of lactation or duration of the feeding [24,25,26], maternal diet, supplementation, and nutritional status [27,28,29,30,31,32,33,34,35] have been demonstrated. In recent years, association studies have found other potential factors influencing the concentrations of vitamins in breast milk, including maternal socio-economic [36,37,38], obstetric, or physiological factors [24,35,36,37,39,40]. Indeed, maternal socio-economic and obstetric factors are changing in China, such as the rate of cesarean delivery, which increased from 3.4% in 1988 to 39.3% in 2008 [41] and to 54.5% in 2011 [42]; as well as the increase in inappropriate gestational weight gain (GWG). The latter is partly due to over nutrition and the rise of different dietary habits [43], which may have an impact on the micronutrient status of lactating women and the composition of their milk. Therefore, it is deemed necessary to research the associations of carotenoids and tocopherols in breast milk with maternal characteristics, and obstetric and nutritional factors in China.

The aims of this study were (1) to determine the composition of carotenoids and tocopherols in breast milk from healthy mothers from urban areas of China along lactation (zero to eight months) (2) to evaluate their interregional differences; and (3) to explore associations with nutrient intake. In addition, the associations with maternal socio-economic and obstetric characteristics, such as age, offspring gender, education, household income, delivery mode, body mass index (BMI), and GWG, were investigated. This study is part of the larger initiative Maternal Infant Nutrition Growth (MING) study.

## 2. Materials and Methods

### 2.1. Background of Participants

The Maternal Infant Nutrition Growth (MING) study was a cross-sectional study designed to investigate the dietary and nutritional status of pregnant women, lactating mothers, and young children aged from birth up to three years living in urban areas of China. In addition, the human milk composition of Chinese lactating mothers was characterized. The study was conducted between 2011 and 2012. Three cities (Beijing is located in Northern China; Suzhou: Eastern China; Guangzhou: Southern China) were chosen for the characterization of human milk according to the geographical location and status of economic development. In each city, one grade A hospital and one maternal and child care hospital were randomly selected, and mothers at lactation period 0 to 240 days were randomly selected based on child registration information. Subject in the period 0–5 days were recruited at the grade A hospital, and subjects in the period 6–11 days and 12–30 days were contacted by phone to join the study, whereas other subjects were recruited at the maternal and child care hospital; if participation was dismissed, a replacement was made. Recruitment and milk sampling, as well as baseline data collection, were done on separate days.

A stratified sampling of 540 lactating mothers in 0–4 days, 5–11 days, 12–30 days, 31–60 days, 61–120 days, and 121–240 days *postpartum* was obtained in the MING study. Eligibility criteria included women between 18–45 years of age giving birth to a single, healthy, full-term gestation, and exclusive breastfeeding at least until four months. Exclusion criteria included gestational diabetes, hypertension, cardiac diseases, or acute communicable diseases. Lactating women who had nipple or lacteal gland diseases, had been using hormones in the last three months, experienced postpartum depression, or had insufficient language skills to understand the study questionnaires were also excluded. The study was approved by the Medical Ethics Research Board of Peking University (No. IRB00001052-11042). Written informed consent was obtained from all subjects participating in the study. The study was also registered in ClinicalTrials.gov with the number identifier NCT01971671.

**Figure 1:**
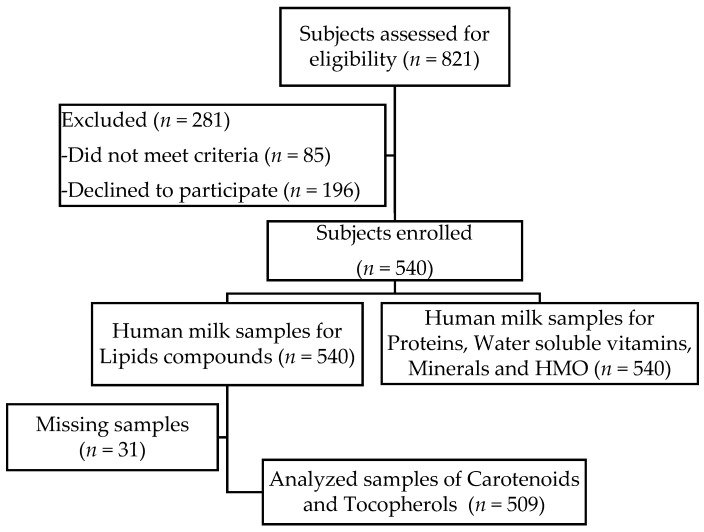
Study flow chart subjects enrolled.

In this cross-sectional study, carotenoids and tocopherols were quantified in 509 breast milk samples collected at different stages from early to late lactation in healthy Chinese women from three different cities (Beijing: *n* = 151; Guangzhou: *n* = 180; Suzhou: *n* = 178). Figure 1 displays the recruitment flowchart from eligibility to sample analysis.

### 2.2. Data Collection

All subjects completed a structured questionnaire, which included socio-economic and lifestyle aspects of the mother such as household income, maternal education, and age. Self-reported weight at the beginning and at the end of pregnancy, the number of gestational weeks at delivery, and delivery mode were also recorded. Additionally, a physical examination evaluated basic anthropometric parameters including height and weight, which were explored to calculate the current body mass index (BMI), and BMI < 18.5, 18.5–24.9, 25.0–29.9, and ≥ 30 kg/m^2^ were defined as underweight, normal weight, overweight, and obese, respectively. These data was obtained to calculate the gestational weight gain (GWG). According to the guidelines from the Institute of Medicines (IOM) in the United States [44], on average underweight women gain 12.5–18 kg, normal weight women gain 11.5–16 kg, overweight women gain 7–11.5 kg, and obese women gain 5–9 kg respectively; inadequate, adequate, and excessive weight gain were confirmed.

Through face-to-face interviews, data collection was performed during the day of the human milk sample collection. Additionally, subjects were contacted by phone and were asked to clarify the date of birth and gender of their baby, since the data was not included in the initial questionnaires.

### 2.3. Dietary Assessment

Dietary intake was assessed using a single 24-h dietary recall. Trained interviewers asked lactating mothers about all foods, beverages, and supplements consumed on the previous day, and then recorded this information in the questionnaire. To estimate the amount of foods and beverages consumed, a picture booklet of common foods consumed in China and measurement aids were used. Details about meals eaten out and food ingredients of homemade foods were also asked and recorded. Additionally, information on the use of dietary supplements was collected, including the name and brand of the supplement, and the amount. After revision of questionnaires, food records were entered in a database, and individual intakes of vitamin A, total carotenoids, retinol, vitamin E, and α-tocopherol were processed with a food composition database created for this study that included data from Chinese Food Composition (CFC) tables 2004 & 2009 [45,46], the Japanese Food Composition (JFC) tables 2005 [47], and branded products and supplements from China. In total, it contained the information of 1773 foods with 36 nutrients. Finally, we also compiled nutritional information from 75 dietary supplements sold in China.

### 2.4. Sample Collection

Breast milk sampling was standardized for all subjects, and was performed in a dim light room in hospitals without direct sunlight exposure. An electric pump (Horigen HNR/X-2108ZB, Xinhe Electrical Apparatuses Co., Ltd., Guangzhou, China) was used to collect the milk. Breasts of the subjects were emptied by the mother herself between 6–7 a.m. After the mothers had breakfast (7–8 a.m.), the samples were collected at the second feeding in the morning (9–11 a.m.) to avoid circadian influence on the outcomes. A single full breast was emptied by trained investigators into a new feeding bottle. We attempted to collect the milk from the same (right) breast, but milk was also collected from the left side when a steady stream of milk was not possible for some mothers. After gently up–down shaking for ~10 times, an aliquot of 15 mL for colostrum and 40 mL for the subsequent time points was secured for characterization purposes. The rest of the milk was returned to the mother for feeding to the infant. Each sample was distributed in 1 mL clear polypropylene tubes under the dim light on ice, labeled with subject information, stored at −80 °C, and then transported to the Nestlé Research Center, Lausanne (Switzerland) for analysis within six months of collection.

### 2.5. Sample Preparation

Briefly, 5 μL of ethanol containing butylated hydroxytoluene (BHT) (79 g/L) and 10 μL of an aqueous solution of deferoxamine mesylate (10 mg/mL) to prevent oxidation, followed by 4 mL methanol, and 1 mL aqueous solution of potassium hydroxide (KOH) (30% *w*/*w*) were added successively to 1 mL of milk into a 15-mL tube. After mixing, the tube was placed for 30 min in a shaking water bath at 37 °C for saponification. The samples were then cooled down on ice, and 5 mL of hexane containing 350 mg/L BHT was added and mixed vigorously for 30 s. Then, the tubes were centrifuged at 2500 rpm for 10 min at 4 °C, and the upper organic phase transferred to a clean 15-mL tube by means of a glass Pasteur pipette. The liquid/liquid extraction process was repeated, and the organic phases combined in the same tube. Once completely dried under nitrogen at room temperature, the residue was dissolved in 70 μL of dioxane/ethanol (1/1, *v*/*v*) to ensure full recovery, and 70 μL acetonitrile were finally added to better match initial mobile phase composition. The samples were centrifuged at 2500 rpm for 10 min at room temperature to clarify extracts, and transferred into adapted low volume Ultra High Performance Liquid Chromatography (UHPLC) vials before analysis. The whole sample preparation and analysis were carried out in a blind laboratory under lights equipped with UV filers to prevent photosensitive analytes from degrading.

### 2.6. Sample Analysis

All the compounds (α-tocopherol, γ-tocopherol, β-carotene, β-cryptoxanthin, lutein, lycopene, and zeaxanthin were determined using a Waters Acquity UHPLC^®^ system (Waters, Milford, MA, USA) equipped with a 2.1 mm × 150 mm Waters Acquity UHPLC^®^ HSS T3 column, 100 Å (particle diameter, 1.8 μm) placed in a column oven set at 35 °C, while autosampler was set at 20 °C.

A 5 μL-aliquot of the final extract was injected into the analytical system. The binary gradient eluting system pumped the mobile phase at a flow rate of 0.4 mL/min. Solvent A was a solution of ammonium acetate 0.05 M in water, and solvent B was a mixture of acetonitrile/diethyl ether/methanol (76/9/15, *w*/*w*/*w*). The eluting gradient program was: 0–20 min, 75% B; 20–22 min, 78% B; 22–22.1 min, 80% B; 22.1–30 min, 100% B; 30–42 min, 100% B; 42–42.1 min, 75% B; 42.1–55 min, 75% B. Quantification was performed by external calibration using pure standards. Concentration of standards was determined by spectrophotometry with corrections made for chromatographic purity. Carotenoids were detected and quantified using ultra violet (UV) at different wavelengths (lycopene, 472 nm; β-carotene, β-cryptoxanthin, lutein, and zeaxanthin, 450 nm; reference wavelength: 560 ± 80 nm), while α-tocopherol and γ-tocopherol were detected and quantified by fluorescence (λ_excitation_: 298 nm, λ_emission_: 328 nm). The standard calibration curve for each compound was constructed by plotting the response (peak area) versus the concentration using a weighted linear regression model.

A quality control (QC) sample consisting in pooled human milk with established analyte concentrations was analyzed in duplicate every 14 samples during analysis campaign. A typical series of analysis included 28 samples. Each analytical series was validated by evaluating accuracy on each QC if at least 67% of QC samples were within 20% of their respective nominal values.

Method performance was evaluated during method validation by analyzing pooled breast milk spiked with standard mixtures in duplicate (*k* = 2) on six different days (*n* = 6). Statistical analysis showed good intra-day and inter-day variability, being both below 15% (expressed as the coefficient of variation). Average recovery rates were respectively 90.0%, 94.0%, 93.3%, 73.3%, and 75.6% for β-carotene, lycopene, β-cryptoxanthin, lutein, and zeaxanthin, and 70.7% and 89.9% for γ-tocopherol and α-tocopherol.

### 2.7. Statistical Analysis

The database was established by using Epi Data version 3.0, and a double data entry was carried out. For the information of demographic characteristics, the data were presented as count with percentage for categorical data, and median with interquartile range for continuous data with non-normal distribution. Before the progress of data analysis, the Shapiro–Wilk test was used to determine whether carotenoids and tocopherols in breast milk, and vitamin intake had a normal distribution or not. Due to non-normal distributions, median values (interquartile range) were performed and ln transformations were applied when doing covariance analysis, multivariate analysis, and correlation analysis. Differences in breast milk vitamins were compared between stages of lactating periods (0–4 days, 5–11 days, 12–30 days, 31–60 days, 61–120 days, and 121–240 days *postpartum*) and cities (Beijing, Suzhou, and Guangzhou cities) by using a nonparametric Kruskal–Wallis test; then, nonparametric Mann–Whitney U tests were employed to detect specific differences between the abovementioned groups further. According to the demographic characteristics of lactating women and their offspring, comparisons in carotenoids and tocopherols concentration were carried out by using covariance analysis models adjusted according to stages of lactation and research cities. Furthermore, multivariate linear regression models were explored to research the demographic influencing factors of carotenoids and tocopherols concentrations in breast milk. To research the correlations between these contents in breast milk and dietary vitamins intake, partial correlation adjusted with stages of lactation and research cities were performed. All of the analyses were carried out using the Statistical Package for the Social Sciences (SPSS Inc., Chicago, IL, USA) version 20.0, and all tests were two-tailed, with statistical significance set at *p* < 0.05.

## 3. Results

The socio-economic characteristics of the lactating women are summarized in Table 1. The mean age of the lactating women was 27.4 ± 4.0 years. The majority of lactating women were unemployed, had completed high school, and had a monthly household income representative of urban China. Although the majority of women had a normal BMI at present, 44% of them had excessive GWG, and up to 48% lactating women had a cesarean delivery. Based on the stage of lactating period, there were no significant differences in the socio-economic characteristics of the lactating women such as age, offspring gender, family’s per capita income, current BMI, GWG, and pregnancy duration. However, it was found that less lactating women during 121–240 days *postpartum* had college education or higher when compared with the others (*p* < 0.05). Meanwhile, more lactating women during 0–4 days and 31–60 days *postpartum* underwent cesarean delivery when compared with those women during 5–11 days, 61–120 days, and 121–240 days *postpartum* (*p* < 0.05), and more lactating women during 31–60 days, 61–120 days, and 121–240 days *postpartum* received dietary supplements than those women during 0–4 days *postpartum* (*p* < 0.05).

The concentrations of the different compounds studied at different periods of lactation are shown in Table 2. As expected, significant differences according to the different periods of lactation were observed for many compounds (*p* < 0.001). The concentrations of most of them (except for lutein, zeaxanthin, and γ-tocopherol) in milk from lactating women during 0–4 days *postpartum* were significantly higher compared with those during other periods (*p* < 0.01); a decrease was observed with the advancement of lactation until it reached stable levels from 12 days *postpartum*. Furthermore, lutein concentrations in milk from lactating women during 0–4 days and 5–11 days *postpartum* were significantly higher compared with those during other periods (*p* < 0.01), while zeaxanthin and γ-tocopherol concentrations remained stable over time.

Carotenoids and tocopherol concentrations in breast milk from lactating women in the three cities are shown in Table 3. The lycopene content in milk from Guangzhou city was significantly higher compared with those from Beijing and Suzhou cities (*p* < 0.001). Similarly, the majority of carotenoids (β-carotene, β-cryptoxanthin, lutein, and zeaxanthin) and α-tocopherol concentrations in milk from Beijing were significantly lower than those from Suzhou and Guangzhou (*p* < 0.01). Meanwhile, γ-tocopherol concentrations in mothers from Suzhou were the highest among the three cities (*p* < 0.001).

Comparisons of the concentrations by characteristics of lactating women and their offspring are provided in Table 4 and supplementary material Table S1. There were no significant associations detected between maternal age, offspring gender, household income, maternal GWG, dietary supplement intake, and tocopherols in breast milk (*p* > 0.05). However, zeaxanthin concentrations in lactating women with vaginal delivery were significant higher compared with those with cesarean delivery (*p* < 0.05). In addition, zeaxanthin concentrations in women with a college education level or above were significant lower than in women with a middle school education level or below (*p* < 0.01). Besides, associations were found between β-carotene and zeaxanthin concentrations and maternal BMI, which indicated that β-carotene concentrations in lactating women with normal BMI were higher than those in overweight women, while zeaxanthin concentrations in milk from underweight lactating women were higher than those from mothers with a normal BMI.

The results from a 24-h food intake recall showed that dietary vitamin A and total carotenoids were not associated with all of the carotenoids in breast milk when adjusted with different cities and lactation period (*p* > 0.05) (Table 5). Similarly, no significant associations were found between vitamin E and α-tocopherol intake, and α- and γ-tocopherol in human milk (*p* > 0.05).

## 4. Discussion

According to the literature [26,35,48,49], colostrum is the first milk secretion after delivery, persisting until the seventh or 10th day *postpartum*; it is followed by the secretion of transitional milk from around the eight and up to 15th day *postpartum*; from then, mature milk is secreted, which shows a relative stable composition. Considering that compositions in human milk do not change abruptly, and the timing varies from one mother to another, there are no clear boundaries among colostrum, transitional milk, and mature milk [50,51,52]. Therefore, we collected human milk during consecutive lactation stages including 0–4 days, 5–11 days, 12–30 days, 31–60 days, 61–120 days, and 121–240 days *postpartum* to characterize carotenoids and tocopherols in human milk along lactation. In the present study, due to the nature of its design, it was difficult to exactly classify the human milk collected within 5–11 days and 12–30 days *postpartum* as either colostrum or transitional milk. On the contrary, human milk collected within 0–4 days and 31–240 days *postpartum* was clearly classified as colostrum and mature milk, respectively.

The highest concentrations of carotenoids and tocopherols were found in colostrum, after that, the concentrations of the different compounds observed in milk at 12–30 days *postpartum* were close to those collected in 30–240 days *postpartum*, which may mean that the changes on carotenoids and tocopherols milk composition gradually slow down until reaching a relatively stable level after 12 days *postpartum*. In accordance with previous studies [26,48,49,53], the levels of most of the compounds (β-carotene, β-cryptoxanthin, lutein, lycopene, and α-tocopherol), except for zeaxanthin and γ-tocopherol, decreased along with lactating stage. The evolution trend of γ-tocopherol concentrations in our study were generally comparable with those in Japan [53] (0.111 ± 0.048 mg/100 mL in 6–10 days *postpartum*; 0.155 ± 0.126 mg/100 mL in 11–20 days *postpartum*, 0.105 ± 0.059 mg/100 mL in 21–89 days *postpartum*; 0.120 ± 0.046 mg/100 mL in 90–180 days *postpartum*; 0.086 ± 0.043 mg/100 mL in 6–10 days *postpartum*).

The carotenoid content in human milk has been studied in several multinational studies, but very few studies report data for a large lactation period (0–240 days *postpartum*). Canfield et al. [21] assessed the levels of carotenoids in human milk from nine countries (five in Asia or the Pacific Rim, three in the Americas, and one in Europe), and found that the concentrations varied greatly among the countries, with only moderate disparities in β-carotene. Regional variability was also found in the longitudinal study of Lipkie et al. [23]. In the present study, we found the median β-carotene concentration in mature milk to be in the same range as that found in Australian, Canadian, Chilean, Japanese, Mexican, Filipinos, English, American, and German mothers in several studies [21,25,26,54]; while the median β-carotene concentrations in colostrum milk were lower than those reported in Germany [25] or Japan [54]. Regarding the relative distribution of carotenoids in Chinese milk, lutein was found to be the major component in mature human milk, which is in agreement with data published by Lipkie et al. [23] and Canfield et al. [21]. On the contrary, β-carotene was the major carotenoid in colostrum, in agreement with a previous longitudinal study in the United States by Song et al. [55].

Differences in carotenoid content of the milk from the three different cities were also observed; milk from Beijing contained significantly lower amounts than milk from Guangzhou or Suzhou for most of carotenoids; while the concentration of lycopene in samples from Guangzhou was higher than the other cities. Taking into consideration that all of the samples were collected with the same protocol and analyzed by the same laboratory, it seems reasonable to believe that the main reason for this difference are environmental factors such as the lower consumption of carotenoid-rich vegetables and fruits. However, more research is needed to confirm the regional differences and elucidate their potential mechanisms.

Median α-tocopherol concentrations (211 μg/100 mL) in Chinese mature milk were lower than those found by researchers in Germany [26], Greece [56], Turkey [36], Poland [49], Canada [57], and Japan [53]. In general, the α-tocopherol content in Chinese milk, not only in colostrum but also in mature milk, was lower than those in industrialized countries, as well as with non-industrialized countries. The large inter-subject variation might be primarily due to dietary habits, the use of dietary supplements, food fortification, or genetic differences among different ethnicities; or to methodological factors, such as the postpartum date of collection, collection of foremilk or hindmilk, or the collection from a single breast of from both breasts.

To date, there is not much evidence of the tocopherols concentration in Chinese human milk. In our study, the median colostrum α-tocopherol concentrations (645 μg/100 mL) were similar to those reported in different groups of Chinese lactating women [58,59] and Polish women [48], but lower than those from German [26] and Brazilian mothers [60], and higher than those of Tunisian [39], Japanese [53], and Inner Mongolia mothers in China [22]. Our results in mature milk (30–240 days *postpartum*) align well to published data [22,25,26,36,38,48,53,56,57]. Intra-country variability was also found, with the highest levels of tocopherols (26.8 mg α-tocopherol) found in mothers from Suzhou. Environmental factors such as dietary intake are likely the reason for this. The intake of 4 mg/day of vitamin E (α-tocopherol) for infants aged less than six months is recommended based on a value of adequate intake (AI) in the Dietary Reference Intakes (DRIs) [61]. According to the recommendations of the Institute of Medicine (IOM) [62], we estimated the supply of vitamin E for infants in the present study using the average quantity of milk consumed by infants aged 0–6 m (780 mL), and found that the possible effect of these milks offered to the infants was 5.0, 3.0, 1.9, 1.6, 1.7, 1.6 mg/day of vitamin E, respectively. Our findings suggested that only colostrum reached an AI proposal for the infant aged less than six months; therefore, the implementation of procedures to increase vitamin E concentrations in milk would be important.

Some nutritional, obstetric, and socio-economic factors have been implicated as being associated with the vitamin A and E concentrations in milk [36,39,60]. In our study, there were no associations detected between the contents of β-cryptoxanthin, lutein, lycopene, α-tocopherol, and γ-tocopherol, and the socio-economic characteristics of women and their offspring, such as maternal age, household income, maternal GWG, or supplement intake. This data provides evidence to suggest that the concentrations of these compounds in breast milk are independent of socio-economic conditions and nutrition in pregnancy. On the contrary, we found associations between the human milk β-carotene and zeaxanthin concentrations, and current BMI. These inverse correlations between some of the carotenoids and current BMI may be due to the underlying mechanism that an excess of body fat increases the consumption of all antioxidant elements in the diet [63], so that lactating women with a higher BMI and more body fat consumed more vitamins A and E than those with lower BMI, resulting in lower carotenoids in human milk. Our results were similar with the previous finding [37] that the percentage of body fat in the lactating women was negatively associated to the concentration of vitamin A in breast milk. In addition, maternal education presented an inverse relationship with lower median concentrations of zeaxanthin among women with high levels of education. Mothers undergoing cesarean delivery presented lower zeaxanthin levels in human milk. Previous studies have associated cesarean delivery with lower colostrum protein content [64] and decreased oxidative stress in colostrum [65], suggesting that cesarean delivery may be detrimental for human milk. Considering that carotenoids contribute to the total anti-oxidative effect of human milk, this relationship of carotenoids and delivery mode requires further investigation to elucidate the possible causal pathways of these mechanisms including organismal regulation, nutrition, and environment.

Previous studies [36,37,66] suggested vitamins A and E in human milk were associated with maternal stores, dietary supplements, fortified foods, and dietary intake. However, our results indicated that neither vitamin A nor E intakes from one 24-h dietary recall was associated with vitamin A and E in human milk, which may be due to the inherent intake variability of one single 24-h dietary recall questionnaire, which did not allow for estimations of an individual’s usual diet, and therefore, it is likely to under- or overestimate some nutrient intakes. Moreover, Jiang et al. [67] found no significant correlation between dietary constituents and α-tocopherol, which is in line with our findings.

There were some limitations to the present study. Firstly, nutrient intake determined by one 24-h recall may introduce some bias by under- or overestimating long-term dietary habits. This variability may result in difficulties accurately estimating individual’s intake when compared with three days dietary recall. Secondly, little is known about the levels of vitamins A and E in maternal plasma associated with the corresponding milk, which would be better than dietary intake to assess maternal nutrient status, due to the impossibility to collect such samples in our research. Thirdly, since our design was a cross-sectional study, we could not collect direct evidence about changes in vitamins A and E with lactation stages. These points should be addressed in future studies. Fourthly, although strict inclusion criteria and exclusion criteria were implemented, we could not ensure that the milk in our study population was sufficient to provide the needed amounts of these compounds. Indeed, our results showed that all of these milks, except for colostrum, did not meet an infant’s nutritional requirement of vitamin E. However, we could not estimate the possible supply of vitamin A to the infant in the present study, due to the lacking detection of retinol in human milk.

## 5. Conclusions

The total concentrations of carotenoids (β-carotene and β-cryptoxanthin, lutein, lycopene, and zeaxanthin) and vitamin E (as α- and γ-tocopherol) were studied in human milk from healthy Chinese women. In summary, our results agreed with previous studies, and suggested that the stage of lactation, regional differences, and obstetric and socio-economic factors might have an effect on the human milk concentrations of carotenoids and tocopherol in healthy Chinese mothers. In view of the great importance of these compounds in human milk to ensure the optimal growth and development of infants, research should continue to explore the biological significance of such results, and improve knowledge on the unique composition of human milk.

## Figures and Tables

**Table 1:** Demographic characteristics of lactating mothers according to different lactating stages.

	0–4 Days (*n* = 77)	5–11 Days (*n* = 89)	12–30 Days (*n* = 73)	31–60 Days (*n* = 90)	61–120 Days (*n* = 90)	121–240 Days (*n* = 90)	*p*-Value
Age, years ^1^							0.097
<25	22 (28.6)	27 (30.3)	26 (35.6)	18 (20.0)	26 (28.9)	36 (40.0)	
25–30	35 (45.5)	41 (46.1)	29 (39.7)	44 (48.9)	50 (55.6)	39 (43.3)	
>30	20 (26.0)	21 (23.6)	18 (24.7)	28 (31.1)	14 (15.6)	15 (16.7)	
Offspring gender ^1^							0.158
Male	35 (45.5)	51 (57.3)	39 (53.4)	48 (53.3)	54 (60.0)	43 (47.8)	
Female	42 (54.5)	38 (42.7)	31 (42.5)	39 (43.3)	36 (40.0)	44 (48.9)	
Education ^1^							0.003 *
Middle school or below	17 (22.1)	12 (13.5)	16 (21.9)	26 (28.9)	22 (24.4)	39 (43.3)	
High school	23 (29.9)	31 (34.8)	27 (37.0)	22 (24.4)	25 (27.8)	23 (25.6)	
College or above	36 (46.8)	45 (50.6)	29 (39.7)	42 (46.7)	41 (45.6)	26 (28.9)	
Family’s per capita income, Yuan/month ^1^							0.140
<2000	16 (20.8)	19 (21.3)	16 (21.9)	24 (26.7)	26 (28.9)	31 (34.4)	
2000–4000	30 (39.0)	37 (41.6)	34 (46.6)	41 (45.6)	40 (44.4)	41 (45.6)	
>4000	27 (35.1)	30 (33.7)	17 (23.3)	23 (25.6)	22 (24.4)	18 (20.0)	
Unclear	4 (5.2)	3 (3.4)	6 (8.2)	2 (2.2)	2 (2.2)	0 (0.0)	
Delivery mode ^1^							0.002 *
Vaginal delivery	29 (37.7)	50 (56.2)	35 (47.9)	37 (41.1)	55 (61.1)	55 (61.1)	
Cesarean delivery	48 (62.3)	37 (41.6)	38 (52.1)	53 (58.9)	35 (38.9)	34 (37.8)	
Present BMI ^1^							0.075
Underweight	1 (1.3)	5 (5.6)	2 (2.7)	2 (2.2)	4 (4.4)	8 (8.9)	
Normal	48 (62.3)	54 (60.7)	47 (64.4)	57 (63.3)	69 (76.7)	65 (72.2)	
Overweight	24 (31.2)	26 (29.2)	23 (31.5)	26 (28.9)	16 (17.8)	16 (17.8)	
Obesity	4 (5.2)	3 (3.4)	1 (1.4)	5 (5.6)	1 (1.1)	1 (1.1)	
Gestational weight gain ^1^							0.300
Inadequate	17 (22.1)	11 (12.4)	14 (19.2)	17 (18.9)	19 (21.1)	26 (28.9)	
Adequate	27 (35.1)	29 (32.6)	28 (38.4)	32 (35.6)	36 (40.0)	25 (27.8)	
Excessive	33 (42.9)	48 (53.9)	29 (39.7)	41 (45.6)	34 (37.8)	39 (43.3)	
Dietary supplements intake ^1^							0.028 *
Yes	5 (6.5)	13 (14.6)	17 (23.3)	17 (18.9)	22 (24.4)	13 (14.4)	
No	72 (93.5)	76 (85.4)	56 (76.7)	73 (81.1)	68 (75.6)	77 (85.6)	
Pregnancy duration, weeks ^2^	39 (38–40)	39 (39–40)	39 (38–40)	39 (38–40)	39.5 (39–40)	40 (39–40)	0.332

BMI, body mass index, was calculated as body weight by height squared (kg/m^2^). Data are expressed as medians (interquartile ranges) for continuous variables and count (percentage) for categorical variables. * Indicates a significant difference among six stages of lactating period (*p* < 0.05). ^1^ Compared by Kruskal–Wallis test; ^2^ Compared by chi-square test.

**Table 2:** Carotenoids and tocopherols concentrations in human milk at different lactation stages (μg/100 mL).

	0–4 Days (*n* = 77)	5–11 Days (*n* = 89)	12–30 Days (*n* = 73)	31–60 Days (*n* = 90)	61–120 Days (*n* = 90)	121–240 Days (*n* = 90)	*p*-Value ^1^	*Post hoc* Test ^2^
β-carotene	8.0 (4.7–15.2)	2.8 (2.0–4.4)	2.1 (1.4–3.1)	1.7 (1.3–3.0)	1.9 (1.4–2.7)	1.8 (1.4–2.6)	<0.001 *	P1 > P2 > P3 = P4 = P5 = P6
β-cryptoxanthin	6.2 (2.4–12.9)	3.4 (1.7–5.7)	2.4 (1.1–3.9)	1.7 (1.1–2.6)	1.8 (1.0–4.0)	2.1 (1.1–3.7)	<0.001 *	P1 > P2 > P3 = P4 = P5 = P6
Lutein	5.7 (2.9–10.2)	7.0 (4.6–10.3)	2.2 (1.2–6.3)	2.9 (0.9–5.9)	2.8 (1.2–6.5)	3.7 (2.4–5.9)	<0.001 *	P1 = P2 > P3 =P4 = P5 = P6
Lycopene	6.3 (4.0–9.9)	2.5 (1.7–4.3)	1.8 (1.2–2.6)	1.4 (1.1–2.0)	1.4 (1.0–2.0)	1.5 (1.3–2.0)	<0.001 *	P1 > P2 > P3 = P4 = P5 = P6
Zeaxanthin	1.0 (0.6–1.5)	1.4 (1.0–2.2)	0.8 (0.4–1.5)	0.8 (0.4–1.4)	1.0 (0.4–1.4)	1.1 (0.8–1.4)	<0.001 *	P2 > P1 = P3 = P4 = P5 = P6
α-tocopherol	645 (388–1176)	382 (236–551)	239 (145–396)	206 (126–345)	212 (112–300)	211 (135–326)	<0.001 *	P1 > P2 > P3 = P4 = P5 = P6
γ-tocopherol	68 (48–121)	63 (43–103)	70 (39–104)	73 (41–120)	68 (39–112)	88 (56–137)	<0.033 *	P2 = P3 < P6; P1 = P4 = P5 = P6

Data are obtained from all three cities and presented as the medians (interquartile ranges). * Indicates a significant difference among the six periods (*p* < 0.05). ^1^ Compared by Kruskal–Wallis test; ^2^ Compared by Mann–Whitney U test with adjusted alpha value (α’ = 0.01). P1: 0–4 days *postpartum*; P2: 5–11 days *postpartum*; P3: 12–30 days *postpartum*; P4: 31–60 days *postpartum*; P5: 61–120 days *postpartum*; P6: 121–240 days *postpartum*.

**Table 3:** Carotenoids and tocopherols concentration of human milk from different cities (Beijing, Suzhou, and Guangzhou cities) (μg/100 mL).

	Beijing (*n* = 151)	Suzhou (*n* = 180)	Guangzhou (*n* = 178)	*p*-Value ^1^	*Post hoc* Test ^2^
B-carotene	1.7 (1.3–3.2)	2.4 (1.7–4.3)	2.7 (1.7–5.0)	<0.001 *	C1 < C2 = C3
β-cryptoxanthin	1.1 (0.8–2.0)	3.6 (2.1–7.7)	2.8 (1.7–5.2)	<0.001 *	C1 < C3 < C2
Lutein	2.2 (1.0–4.1)	4.9 (2.6–7.9)	5.8 (2.9–8.7)	<0.001 *	C1 < C2 = C3
Lycopene	1.7 (1.3–2.8)	1.7 (1.3–2.7)	2.1 (1.4–3.8)	0.006 *	C1 = C2 < C3
Zeaxanthin	0.8 (0.4–1.4)	1.1 (0.7–2.0)	1.1 (0.7–1.5)	<0.001 *	C1 < C2 = C3
α-tocopherol	215 (117–333)	296 (208–478)	285 (148–479)	<0.001 *	C1 < C2 = C3
γ-tocopherol	71 (48–107)	94 (59–148)	53 (31–88)	<0.001 *	C3 < C1 < C2

Data are obtained from all six lactation stages and presented as the medians (interquartile ranges). * Indicates a significant difference among the three cities (*p* < 0.05). ^1^ Compared by Kruskal–Wallis test; ^2^ Compared by Mann–Whitney U test with adjusted alpha value (α’ = 0.01). C1: Beijing; C2: Suzhou; C3: Guangzhou.

**Table 4:** Comparisons of the carotenoids concentration in human milk by the characteristics of lactating women.

	β-carotene		β-cryptoxanthin		Lutein		Zeaxanthin	
	Adjusted ^1^ β (95% CI)	SEM	Adjusted ^1^ β (95% CI)	SEM	Adjusted ^1^ β (95% CI)	SEM	Adjusted ^1^ β (95% CI)	SEM
Age, years								
<25	−0.05 (−0.18, 0.08)	0.07	0.11 (−0.06, 0.28)	0.09	−0.13 (−0.35, 0.08)	0.11	−0.01 (−0.14, 0.15)	0.07
25–30	Reference		Reference		Reference		Reference	
>30	0.10 (−0.03, 0.23)	0.07	0.11 (−0.07, 0.29)	0.09	−0.03 (−0.15, 0.19)	0.11	0.12 (−0.03, 0.27)	0.08
Education								
Middle school or below	Reference		Reference		Reference		Reference	
High school	0.03 (−0.11, 0.17)	0.07	−0.18 (−0.36, 0.01)	0.09	0.12 (−0.11, 0.35)	0.12	−0.14 (−0.30, 0.02)	0.08
College or above	0.09 (−0.04, 0.23)	0.07	−0.12 (−0.30, 0.06)	0.09	0.08 (−0.14, 0.31)	0.11	−0.15 (−0.31, −0.00) *	0.08
Delivery mode					0			
Vaginal delivery	0.04 (−0.07, 0.15)	0.05	0.03 (−0.11, 0.17)	0.07	0.14 (−0.04, 0.31)	0.09	0.13 (0.02, 0.25) *	0.06
Cesarean delivery	Reference		Reference		Reference		Reference	
Current BMI					0			
Underweight	0.01 (−0.25, 0.26)	0.13	−0.02 (−0.35, 0.32)	0.17	0.32 (−0.09, 0.74)	0.21	0.29 (0.01, 0.57) *	0.14
Normal	Reference		Reference		Reference		Reference	
Overweight	−0.17 (−0.29, −0.05) *	0.06	−0.16 (−0.32, 0.00)	0.08	−0.11 (−0.31, 0.09)	0.10	−0.07 (−0.21, 0.07)	0.07
Obesity	−0.24 (−0.54, 0.07)	0.16	−0.16 (−0.57, 0.24)	0.21	0.16 (−0.34, 0.66)	0.26	−0.18 (−0.52, 0.16)	0.17

CI, confidence interval; SEM, standard error of mean. Multivariate linear regression model considering carotenoids in breast milk as the dependent variable and the other variables studies as independent variables. ^1^ Adjusted for periods of lactation (0–4 days, 5–11 days, 12–30 days, 31–60 days, 61–120 days, and 121–240 days *postpartum*), cities (Beijing, Suzhou, and Guangzhou cities), and other independent influencing factors listed above. * Indicates a significant difference when compared with the reference (*p* < 0.05). β-carotene: R^2^ = 0.482, *p* < 0.001; β-cryptoxanthin: R^2^ = 0.366, *p* < 0.001; Lutein: R^2^ = 0.282, *p* < 0.001; Zeaxanthin: R^2^ = 0.124, *p* < 0.001.

**Table 5:** The associations between vitamins intake and concentrations of carotenoids and tocopherols in breast milk.

		β-carotene	β-cryptoxanthin	Lutein	Lycopene	Zeaxanthin	α-tocopherol	γ-tocopherol
Dietary intake of vitamin A	*R*	0.022	0.026	0.027	−0.007	0.075	-	-
	*p* ^1^	0.618	0.562	0.537	0.881	0.093	-	-
Dietary intake of total carotenoids	*R*	0.055	0.002	0.007	−0.038	0.003	-	-
	*p* ^1^	0.220	0.963	0.880	0.398	0.948	-	-
Dietary intake of vitamin E	*R*	-	-	-	-	-	−0.083	0.006
	*p* ^1^	-	-	-	-	-	0.063	0.885
Dietary intake of α-tocopherol	*R*	-	-	-	-	-	−0.033	−0.084
	*p* ^1^	-	-	-	-	-	0.456	0.058

^1^ Partial correlation was performed to analyze the correlations adjusted with cities (Beijing, Suzhou, and Guangzhou cities) and periods of lactating (0–4 days, 5–11 days, 12–30 days, 31–60 days, 61–120 days, and 121–240 days *postpartum*).

## Supplementary Materials

The following are available online at www.mdpi.com/2072-6643/9/11/1229/s1, Table S1: Comparisons of carotenoids and tocopherols concentration according to characteristics of lactating women and their offspring (μg/100 mL).
