# Supplementary material for: Concentrations of Carotenoids and Tocopherols in Breast Milk from Urban Chinese Mothers and Their Associations with Maternal Characteristics: A Cross-Sectional Study
Source: Nutrients. 2017 Nov 9;9(11):1229. doi: 10.3390/nu9111229 (PMC5707701; doi:10.3390/nu9111229)
Supplement: Supplementary file 1 [file nutrients-09-01229-s001.zip › nutrients-235171-supplementary.pdf]

Table S1. Comparisons of carotenoids and tocopherols concentration according to characteristics of lactating women and their offspring (μg/100mL).

	β-carotene [1]	β-cryptoxanthin [1]	Lutein [1]	Lycopene [1]	Zeaxanthin [1]	α-tocopherol [1]	γ-tocopherol [1]
Age, years							
< 25	2.4 (1.7-3.9)	3.1 (1.7-5.7) [a]	4.2 (2.2-7.3)	1.8 (1.3-3.2)	1.1 (0.7-1.8)	284 (175-452)	77 (52-131)
25-30	2.2 (1.4-3.9)	2.1 (1.1-4.0) [a]	4.1 (2.0-7.0)	1.8 (1.3-2.8)	1.0 (0.6-1.4)	261 (139-403)	67 (42-107)
> 30	2.5 (1.6-5.0)	2.5 (1.1-4.7)	3.9 (1.8-8.7)	1.8 (1.3-2.9)	1.1 (0.7-1.6)	281 (179-473)	73 (41-120)
Offspring gender							
Male	2.4 (1.5-4.2)	2.5 (1.4-4.8)	4.6 (2.2-8.0)	1.8 (1.3-3.2)	1.1 (0.6-1.7)	274 (153-452)	73 (44-121)
Female	2.2 (1.5-4.2)	2.1 (1.1-4.6)	3.6 (1.7-6.8)	1.8 (1.3-2.8)	1.0 (0.6-1.5)	275 (171-442)	68 (44-107)
Education							
Middle school or blow	2.3 (1.5-4.1)	2.7 (1.5-5.2) [a]	4.1 (2.1-7.4)	1.7 (1.3-2.4)	1.1 (0.7-1.8) [a]	283 (165-448)	84 (45-143)
High school	2.2 (1.5-4.1)	2.4 (1.4-4.8)	4.5 (2.0-7.1)	1.8 (1.1-3.1)	1.1 (0.6-1.5)	263 (161-419)	67 (43-103)
College or above	2.4 (1.5-4.9)	2.1 (1.0-4.5) [a]	3.9 (2.0-7.3)	2.0 (1.3-3.2)	1.0 (0.6-1.4) [a]	273 (156-468)	70 (44-107)
Family's per capita income, Yuan/mo							
< 2000	2.2 (1.4-3.9)	2.5 (1.4-5.1)	3.8 (2.1-7.4)	1.7 (1.3-2.8)	1.0 (0.6-1.5)	268 (164-451)	76 (46-122)
2000-4000	2.2 (1.4-4.1)	2.1 (1.1-4.1)	3.8 (1.8-6.2)	1.7 (1.3-2.8)	1.0 (0.6-1.5)	272 (142-426)	73 (43-112)
> 4000	2.5 (1.7-4.8)	2.5 (1.4-5.8)	5.3 (2.1-8.7)	2.2 (1.4-3.4)	1.1 (0.6-2.0)	293 (174-496)	67 (43-109)
Unknown	2.2 (1.7-5.9)	1.7 (1.2-5.3)	5.7 (2.3-8.8)	2.0 (1.3-3.5)	0.8 (0.6-1.9)	283 (178-432)	58 (28-130)
Delivery mode							
Vaginal delivery	2.4 (1.5-4.1)	2.7 (1.4-4.8)	4.6 (2.2-7.5) [a]	1.8 (1.3-2.7)	1.1 (0.7-1.7) [a]	274 (164-453)	71 (44-113)
Cesarean delivery	2.2 (1.5-4.3)	2.1 (1.1-4.7)	3.5 (1.7-6.9) [a]	1.8 (1.3-3.5)	1.0 (0.6-1.4) [a]	274 (161-427)	73 (44-115)

Present BMI							
Underweight	2.6 (1.6-3.8)	2.8 (1.5-4.0)	7.4 (2.9-9.0) [a]	1.5 (1.1-2.1)	1.5 (0.8-2.6) [abc]	285 (214-463)	80 (47-122)
Normal	2.4 (1.5-4.5) [a]	2.5 (1.4-5.3)	4.3 (2.1-7.4)	1.8 (1.3-2.9)	1.1 (0.6-1.5) [a]	268 (161-438)	68 (41-112)
Overweight	2.0 (1.4-3.9) [ab]	1.8 (1.1-3.9)	3.4 (1.5-6.4) [a]	2.0 (1.3-3.2)	1.0 (0.6-1.5) [b]	274 (158-446)	75 (51-121)
Obesity	1.7 (1.1-2.2) [b]	1.4 (0.8-5.6)	3.4 (1.3-6.9)	2.1 (1.3-4.6)	0.7 (0.4-1.4) [c]	288 (141-603)	93 (62-180)
Gestational weight gain							
Inadequate	2.4 (1.5-5.3)	2.8 (1.5-4.7)	5.1 (2.3-8.6)	1.7 (1.3-2.7)	1.3 (0.7-1.8)	279 (151-449)	67 (41-106)
Adequate	2.4 (1.5-4.5)	2.5 (1.3-6.0)	4.5 (2.1-8.1)	2.0 (1.3-3.3)	1.1 (0.6-1.6)	282 (171-455)	69 (42-118)
Excessive	2.2 (1.5-4.0)	2.1 (1.0-4.2)	3.5 (1.8-6.4)	1.8 (1.3-2.9)	1.0 (0.6-1.4)	265 (158-426)	76 (48-112)
Dietary supplements intake							
Yes	2.2 (1.7-3.6)	2.4 (1.1-3.8)	3.9 (2.1-6.6)	1.7 (1.3-2.5)	1.1 (0.6-1.4)	274 (146-381)	68 (35-107)
No	2.3 (1.5-4.4)	2.4 (1.3-4.8)	4.2 (2.1-7.5)	1.8 (1.3-3.2)	1.0 (0.6-1.7)	273 (162-457)	72 (45-116)

Data are presented as the medians (interquartile ranges). [1] All of data were performed ln transformations and then compared by analysis covariance adjusted with cities (Beijing, Suzhou, and Guangzhou cities) and periods of lactating (0-4 d, 5-11 d, 12-30 d, 31-60 d, 61-120 d, and 121-240 d *postpartum*). Median values in the same column with same superscript letters (a, b, c) were significantly different, $p < 0.05$.
